# The Association of *TP53*, *BCL2*, *BAX* and *NOXA* SNPs and Laryngeal Squamous Cell Carcinoma Development

**DOI:** 10.3390/ijms252111849

**Published:** 2024-11-04

**Authors:** Tomas Jakstas, Agne Bartnykaite, Evaldas Padervinskis, Aurelija Vegiene, Elona Juozaityte, Virgilijus Uloza, Rasa Ugenskiene

**Affiliations:** 1 Department of Otorhinolaryngology, Lithuanian University of Health Sciences, 50161 Kaunas, Lithuania; tomas.jakstas@lsmu.lt (T.J.); evaldas.padervinskis@lsmu.lt (E.P.); aurelija.vegiene@lsmu.lt (A.V.); 2 Oncology Institute, Lithuanian University of Health Sciences, 50161 Kaunas, Lithuania; agne.bartnykaite@lsmu.lt (A.B.); elona.juozaityte@lsmu.lt (E.J.); 3 Oncology Research Laboratory, Oncology Institute, Lithuanian University of Health Sciences, 50161 Kaunas, Lithuania; rasa.ugenskiene@lsmu.lt; 4 Department of Genetics and Molecular Medicine, Lithuanian University of Health Sciences, 50161 Kaunas, Lithuania

**Keywords:** SNP, *TP53*, *BCL2*, *NOXA*, *BAX*, laryngeal squamous cell carcinoma

## Abstract

Head and neck cancer is the seventh leading cancer diagnosis worldwide. One of the most common cancers in the head and neck region is laryngeal cancer. In past years, the incidence of laryngeal squamous cell carcinoma has risen by 23%, and despite progress in treatment modalities, the survival rate has not changed. It is well known that genetic alterations may contribute to individuals’ susceptibility to cancer. Research of genetic alterations, such as single nucleotide polymorphisms, is essential to understanding carcinogenesis and susceptibility of laryngeal squamous cell carcinoma. A total of 200 LSCC patients and 200 controls were included in this retrospective case-control study; both groups were matched by age and sex. In the present study, we analyzed six SNPs in genes essential for apoptosis regulation: *TP53* (rs9895829, rs17884306), *BCL2* (rs1564483, rs4987855), *BAX* (rs704243), *NOXA* (*PMAIP1*) (rs1041978, rs78800940). We evaluated their associations with the risk of LSCC development, its pathomorphological manifestation, and patients’ overall survival rate. Genotyping was carried out using RT-PCR. The AG genotype of rs9895829 was more prevalent in controls than in cancer patients, leading to lower susceptibility to LSCC (OR = 0.301; 95%CI 0.096–0.940; *p* = 0.039). None of the analyzed SNPs showed an association with pathomorphological features of LSCC, but *NOXA* rs1041978 T allele carriers were found to be diagnosed with LSCC at an older age (OR = 1.962; 95%CI 1.072–3.592; *p* = 0.031). There was no statistically significant association between investigated SNPs and patient OS. The present study indicates that the AG genotype of rs9895829 provides a protective effect against LSCC development.

## 1. Introduction

Head and neck cancer (HNC) is the seventh leading cancer diagnosis worldwide [1]. This tumor involves cancers of the nasopharynx, oropharynx, hypopharynx, larynx, salivary glands, lip, oral cavity, and paranasal sinuses, all with distinct presentation, clinical course, and outcome. Most of the published studies include these cancers under the general term of head and neck cancer despite their differences. According to various literature sources, laryngeal cancer makes up approximately one-third of head and neck cancers [2] and is one of the most common cancers in the head and neck region. Laryngeal squamous cell carcinoma (LSCC) is the most common histological type of laryngeal cancer, as other types represent only 5% of laryngeal cancers [3]. In the past decades, the incidence of LSCC has risen by 23% [1,4]. New techniques have been implemented to treat LSCC. Minimally invasive techniques with voice preservation have been introduced in LSCC treatment over the last decades, and they are different from previously used total laryngectomy, even in the early stages of the disease. Radiochemotherapeutic modalities have also been developed and implemented. However, despite the new techniques, the outcome in LSCC has not changed in past decades—the survival rate has been relatively high in the early stages (stage I, 89%), though it is notably low in the advanced stages (stage IV, 38%) [5,6].

Even though the main risk factors (smoking, alcohol consumption, and male sex) are previously described in research—there are many unknown factors affecting LSCC development, such as genetics, viral agents, environmental pollutants, changes in the microbiome, and others [7,8,9,10,11]. It is well known that genetic alterations may contribute to an individual’s susceptibility to cancer development. Genetic studies have highlighted genetic alterations that can potentially increase the risk for LSCC [12,13,14,15], however further research is needed to understand the role of the genetic factors in LSCC carcinogenesis.

Single nucleotide polymorphisms (SNPs) are among the most prevalent genetic variations in the human genome. Understanding the role of SNPs leading to increased cancer susceptibility is essential to comprehend the underlying molecular basis of carcinogenesis. Various research highlighted the importance of SNPs as potential diagnostic and therapeutic biomarkers of many cancer types. However, the number of SNPs in the human body reaches over 88 million [16], which makes it a complex, time and finance-consuming task to be solved for researchers. This is why SNPs in genes, which are potentially involved in carcinogenesis mechanisms (e.g., DNA repair, cell cycle regulation, metabolism, immunity), are prioritized for this type of research.

Changes in apoptosis mechanisms have been extensively reported in tumorigenesis [17]. Proteins encoded by the *BCL-2, TP53*, *NOXA,* and *BAX* genes are important regulators of the mitochondrial apoptosis mechanism [18,19,20]. Changes in these genes may lead to alterations in apoptosis, resulting in cancer development and progression [17].

There are very few studies in HNC and no studies investigating LSCC selectively regarding SNPs in *TP53*, *BCL-2*, *NOXA*, and *BAX* genes. Rs9895829 is an intronic variant of *TP53,* potentially interfering with the binding and function of transcriptional factors or affecting splicing. Rs17884306 of *TP53*, along with rs1564483 and rs4987855 of *BCL2*, rs704243 of *BAX*, and rs1041978 of *NOXA*, are all located in the 3′ untranslated regions (3′UTR) of the mentioned genes and have the potential to affect microRNA binding and its function to control gene expression. Rs78800940 is a missense variant of *NOXA,* thus encoding a different amino acid in a corresponding position of the protein with a probability of altered protein function.

Therefore, we aimed to investigate selected SNPs in the *TP53*, *BCL-2*, *NOXA,* and *BAX* genes and their association with LSCC development risk, cancer morphological features, and patient overall survival rate. We hypothesize that specific genotypes or alleles within these genes may contribute to the predisposition for the development of LSCC or enhance protective mechanisms against it.

## 2. Results

The research involved 200 male patients with LSCC, whose median age at diagnosis was 64.5 years (ranging from 49 to 85). Furthermore, 200 healthy male individuals with a median age of 65.5 years (ranging from 50 to 75) were included as controls. After conducting statistical analysis, it was found that there was no significant difference in age between the case and control groups (*p* = 0.073) (Table 1).

The distribution of analyzed SNPs in *TP53* (rs17884306), *BCL2* (rs1564483 and rs4987855), *BAX* (rs704243), and *NOXA* (rs78800940 and 1041978) were in accordance with HWE. Even though rs9895829 of *TP53* did not adhere to HWE, we decided not to exclude it from further study. It was determined that the AG genotype of rs9895829 was more prevalent in controls than in cancer patients, leading to lower susceptibility to laryngeal cancer (OR = 0.301; 95%CI 0.096–0.940; *p* = 0.039). The result remained very similar and statistically significant after adjustment for age (Table 2).

The subsequent analysis of the LSCC study group revealed no statistically significant associations between investigated SNPs and pathomorphological features of LSCC. The median age at the diagnosis was 64.5, according to it the groups were divided into two: ≤65 years and >65 years. *NOXA* rs1041978 T allele carriers were found to be diagnosed with LSCC at an older age (OR = 1.962; 95%CI 1.072–3.592; *p* = 0.031) (Table 3).

Overall survival of LSCC patients was compared by log-rank, Breslow, and Tarone-Ware tests. Survival curves were generated using the Kaplan–Meier method. There were no significant differences in the survival of patients with different genotypes (Supporting Information: Supplementary Figures S1–S7, Supplementary Tables S1–S7). Our data suggests that the analyzed SNPs do not contribute to the overall survival rate of LSCC patients.

## 3. Discussion

The proteins encoded by the *BCL-2*, *TP53*, *NOXA*, and *BAX* genes play a vital role in regulating the mitochondrial apoptosis pathway through their interdependent interactions [18,19,20]. The mitochondrial apoptosis pathway is found to be the most dysregulated mechanism of cell death in cancer [21]. This is why targeting apoptosis activation is one of the main goals of cancer therapy. It is important to note that there are also other types of cell death mechanisms, though it is not yet fully clear whether targeting these mechanisms would benefit cancer treatment. Nonetheless, research aiming to understand carcinogenesis through mitochondrial apoptosis and the SNP of genes responsible for it is fundamental.

*TP53* is one of the most investigated genes in any cancer. Nearly half of all human cancers have been found to contain mutations in the *TP53* gene [22]. Research has shown that mutations in the *TP53* gene are the most frequent of all genetic alterations in HNC and are associated with cancer invasiveness, distant metastasis formation, and cancer proliferation, which leads to higher cancer aggressiveness, thus lowering the survival rate of patients despite treatment [23,24]. Based on different research, mutations in the *TP53* gene in LSCC vary. Barnard et al. (2003) indicated that 25% of the patients had mutations in *TP53* [25], whereas Zhou et al. (2016) found *TP53* to be mutant in 83% of laryngeal and hypopharyngeal cancers [26]. Moreover, Caponio et al. (2020) performed a comprehensive bioinformatic genomic study and evaluated the relation of the location and the type of *TP53* mutation with the clinicopathological characteristics. It was demonstrated that mutations in the different *TP53* subdomains are associated with distinct clinical behaviour of HNSCC and, consequently, have a differential impact on the disease prognosis in HNSCC subgroups (oral cavity, oropharynx, hypopharynx, and larynx) [27]. SNPs in *TP53* have been investigated before, though only a few studies have been done with HNC patients, especially LSCC.

Germline *TP53* rs9895829 has not been previously studied in HNC, so there is a lack of existing research that our data could be compared to with a similar group of subjects. Zhuo-Miao Ye et al. (2020)*,* in their meta-analysis [22], demonstrated that the *TP53* rs9895829 AG genotype showed an association with pancreatic cancer risk. Eiholzer et al. (2020), in their research, investigated 89 patients with glioblastoma, 122 patients with prostate and two breast cancer groups consisting of 75 and 314 patients with three control groups consisting of 599, 355, and 668 individuals. They demonstrated that heterozygotes of rs1042522 in combination with rs9895829 heterozygotes carry a 5.35-fold increased risk of developing cancer [28]. Our research findings suggest that the *TP53* rs9895829 AG genotype lowers the risk of LSCC by more than three times (OR = 0.301; 95%CI 0.096–0.940; *p* = 0.039). These dissimilarities might be due to otherwise different locations of cancers, different cancer histological profile, and their pathogenesis. As squamous cell carcinoma is the most prevalent cancer in the larynx, it is scarce in pancreatic, breast, or prostate cancers [29,30,31]. Although definitive conclusions regarding the influence of the AG genotype on gene function cannot be drawn, it is our assertion that it likely contributes to a stable functional state, as opposed to a state of functional gain or loss. Zhuo-Miao Ye et al. (2020) research [22] also showed a significant association between rs9895829 and the mRNA expression levels of *TP53*. Although our research did not examine mRNA expression, it is an area of focus for our research group in the future.

BCL-2, *NOXA*, and *BAX* proteins all belong to the BCL-2 family that regulates apoptosis by interacting with each other, thus playing an essential role in mitochondrial apoptosis regulation [32,33]. *NOXA* belongs to the pro-apoptotic sub-group of BCL-2 proteins, whereas BCL-2 belongs to the antiapoptotic sub-group, and *BAX* is one of the effectors of apoptosis [32]. Thus, changes in genes regulating the production and activity of these proteins may lead to changes in cell apoptosis mechanisms, leading to cancer development, progression, and resistance to therapy. However, targeting these proteins may provide new cancer treatment modalities. BCL-2 protein expression is found to be elevated in various cancers [33]. It is worth mentioning that SNPs in genes encoding these proteins attracted attention in past studies investigating breast, gastric, lung, or other cancers but have not yet been studied in the LSCC group. Yang et al. (2016) found that the A allele in the *BCL-2* rs2279115 polymorphism had a substantial impact on diminishing small-cell lung cancer risk in Chinese populations [34]. Xu et al. (2013) found that the *BCL2* 3′UTR rs1564483 A allele was associated with a decreased lung cancer risk and better survival for advanced non-small cell lung carcinoma [35]. Jouneghani et al., in their recent (2023) study, investigated rs1564483 SNP and its relationship with breast and gastric cancers. It was found that there was no relevant connection between the studied SNP and breast cancer, though it showed a notable association between the AG genotype and gastric cancer risk [36]. Shi et al. (2012) demonstrated that regional lymph node metastasis is less frequent for patients with minor allele of rs4987855 [37]. However, despite research that was done with other cancers and their findings, we did not observe any statistically significant correlations between investigated rs1564483 and rs4987855 and LSCC. According to a meta-analysis by Silva et al. (2023), Bcl-2 protein overexpression is linked to worse lymph node metastasis, overall survival, and disease-free survival in patients with HNC though, it is important to note that this conclusion may not be entirely reliable due to the variability of included studies [38]. Our study was not designed to assess protein expression levels; however, this represents a potential area of focus for future investigations.

Both *NOXA (PMAIP1*) and *BAX* genes have been studied in other research: Ishida et al. (2008) found that *NOXA (PMAIP1*) may play a significant role in pancreatic cancer progression [39], Hyunhee et al. (2019) found that *NOXA* (*PMAIP1*) was downregulated in non-small lung cancer cells thus leading to cell proliferation and cancer development [40]. *NOXA*(*PMAIP1*) was also identified as a gene that helps to differentiate healthy controls from patients with HNC. Non-invasive saliva testing [41] led to the conclusion that *NOXA (PMAIP1*) plays a vital role in HNC carcinogenesis. Even though our data did not show the association between *NOXA* (*PMAIP1*) and the risk of developing LSCC, we did find that rs1041978 T allele was associated with an older age at LSCC diagnosis (OR = 1.962; 95%CI 1.072–3.592; *p* = 0.029). Higher expression of Bax protein was also a predicting factor for radiosensitivity and better response to various chemotherapeutic drugs for head and neck cancer patients [42,43]. Although Casado et al. (2002) showed that Bax expression did not predict response to induction therapy in locally advanced HNC, their research group involved only 43 patients [44]. Tano et al. (2013) and colleagues discovered that the balance expression ratio of antiapoptotic *Bcl-2* and *Bax* genes in circulating immune cells (peripheral blood mononuclear cells) is highly prognostic for patients with HNC [45]. Though these genetic alterations were earlier analyzed, to the best of our knowledge, SNPs in these genes have not yet been reported in any HNC yet. Therefore, this is the first research on *BAX* rs704243, *NOXA* (*PMAIP1*) rs78800940 and rs1041978 SNPs in HNC or any cancer.

The strength of this study is in collecting a pure LSCC cohort, which is age and sex-matched with the control group and collecting all samples in a single hospital unit using a standardized protocol for sample handling and investigation. The finding that *TP53* rs9895829 AG genotype lowers the risk of LSCC by more than three times is of significant importance, as it is the first study demonstrating this on squamous cell carcinoma in the larynx. Pure cohort studies focusing on single localization of head and neck cancer are essential as they all have different etiology, clinical course, and even distinct genetic predispositions [46,47]. Therefore, single location cancers should be investigated separately to avoid making false assumptions.

There are some limitations to consider in the current study. A larger sample size would benefit conclusions as well as protein expression levels measurements. Environmental factors (e.g., alcohol consumption, smoking habits) could also be included. Longer follow-up time for patients would also benefit conclusions about the patients’ overall survival rate. However, addressing these limitations could be a focus for future research.

## 4. Materials and Methods

This study was designed as a retrospective case-control study. The aim was to investigate the selected SNPs in the *TP53*, *BCL-2*, *NOXA* and *BAX* genes and their association with LSCC development risk, cancer morphological features, and patient overall survival rate.

### 4.1. LSCC Group

All patients included in this study were recruited from 2016 to 2023 at the Department of Otorhinolaryngology, Lithuanian University of Health Sciences (LUHS). LSCC group patients underwent a comprehensive examination of the ear, nose, and throat, which included flexible endoscopy and/or video laryngoscopy. Biopsy was performed during direct microlaryngoscopy for all patients. Histopathologically, LSCC was confirmed by the Department of Pathology, LUHS. The diagnosis of LSCC was determined by analyzing clinical data and the results of histological examination, as well as laryngeal and neck computed tomography (CT) with contrast enhancement or magnetic resonance imaging (MRI). The staging of LSCC was performed following the Guidelines for Head and Neck Cancers Classification, Version 2.2020, which were accepted by the National Comprehensive Cancer Network (NCCN) [48]. Only male patients were included in the study. Patients who were suspected of having other types of cancer or were under 18 years of age were excluded from the study. Clinical information was gathered through personal interviews and by reviewing the case records of patients.

To conduct SNP analyses, peripheral venous blood samples were collected. The LSCC group data about the overall survival rate was collected from the Lithuanian State Register of Death Cases and Their Causes. The range of follow-up time was from 12 to 93 months.

**Figure 1:**
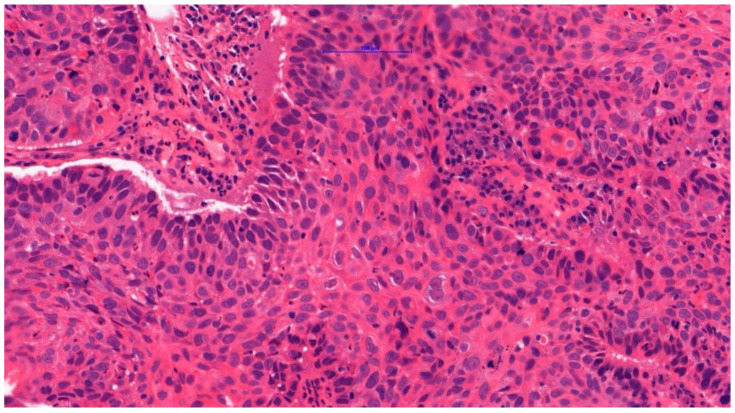
Conventional squamous cell carcinoma. Heterogeneity of tumor cells, abundant mitoses, cornification in the cytoplasm of cells, pronounced infiltration of lymphocytes, plasma cells, and neutrophilic granulocytes observed in the stroma.

This study included a cohort of 200 male individuals diagnosed with LSCC, as LSCC demonstrates a significant male predominance in its epidemiology. Information about tumor size (T), nodal metastasis (N), distant metastasis to other organs (M), clinical stage (ST), and cancer cell differentiation grade (G) was collected. T, N, M stages, ST and G were divided into two groups: T1-2 (low stage) versus (VS) T3-4 (high stage), N0 (lymph nodes without metastasis) VS N1-3 (lymph nodes with metastasis), M0 (no distant metastasis) VS M1 (with distant metastasis), ST 1-2 (early stage) VS ST 3-4 (advanced stage) and G1-2 (well/moderately differentiated cancers) VS G3 (poorly differentiated cancer) [49,50]. Among the 200 cases analyzed, 190 were identified as Conventional Squamous Cell Carcinoma (Figure 1), in accordance with the 5th edition of the WHO classification of head and neck tumors. Consequently, these cases were aggregated and analyzed as a singular group of LSCC.

### 4.2. Reference Group

This study’s reference group comprised 200 male individuals recruited during an annual health checkup at the Department of Family Medicine, LUHS. Patients who had received treatment for cancer or were suspected to have any oncological disease were excluded from the study.

The LSCC patients and reference groups were matched by age and sex (males only) (Table 1). The study was approved by the Kaunas Regional Biomedical Research Ethics Committee (authorization protocols No. BE-2-37, No. BE-2-10, and No. P1-BE-2-10/2014), and informed consent was obtained from all the participants prior to inclusion in the study.

### 4.3. Sample Size

To determine the appropriate sample size for this study, the formula n  =  Z^2^ × p × (1 − p)/E^2^ was used, where n represents the necessary sample size, Z is the 95% confidence level, p is the minimal allele frequency, and E is the margin of error. The minimal allele frequency was obtained from the SNP database and ranged from 5 to 27% in European (1000 Genome Project). The margin of error was set at 8%. Based on these parameters, the sample size was calculated to be between 18 and 105 patients.

### 4.4. Statistical Analysis

To evaluate differences in genotype frequencies among the groups, a chi-square test was used to assess the Hardy-Weinberg equilibrium (HWE) with a *p*-value greater than 0.05. Pearson’s chi-square or Fisher’s exact tests were used to analyze the associations between SNP genotypic/allelic models and the risk of LSCC and pathomorphological tumor characteristics. Univariate logistic regression was used to calculate the odds ratios (ORs) with a 95% confidence interval (CI) to estimate the impact of each SNP on pathomorphological characteristics and determine whether it increases or decreases the risk of developing LSCC. The relationships between the studied SNPs and overall survival (OS) were assessed for survival analysis. The Kaplan-Meier method was used to generate survival curves, and log-rank, Breslow, and Tarone-Ware tests were used to compare them. A value of less than 0.05 was considered statistically significant.

The Statistical analysis was performed using IBM Statistical Package for the Social Sciences (SPSS) version 29.0 (SPSS Inc., Chicago, IL, USA).

### 4.5. Genotyping of SNPs

The genotyping of *TP53* (rs9895829 and rs17884306), *BCL2* (rs1564483 and rs4987855), *BAX* (rs704243), and *NOXA (PMAIP1)* (rs78800940 and rs1041978) was carried out at the Oncology Research Laboratory of Oncology Institute at LUHS. Venous blood samples for DNA extraction were collected in ethylenediamine tetra-acetic acid tubes. According to the manufacturer’s recommendations, genomic DNA from peripheral blood leucocytes was extracted using a DNA purification kit (Thermo Fisher Scientific, Waltham, MA, USA). The SNPs were estimated using TaqMan genotyping assays (Thermo Fisher Scientific Baltics, Vilnius, Lithuania) by QuantStudio 3 Real-Time PCR system. The assays IDs were C__29631782_10, C__60493418_10, C___3170929_10, C__22274072_10, C___1444870_30, C___7905447_1_, C__32863307_10.

## 5. Conclusions

This study presents valuable insight into LSCC carcinogenesis as *TP53* rs9895829 AG genotype was proven to provide a protective effect on LSCC development. None of the analyzed SNPs were associated with pathomorphological features of LSCC, and the genotypic distribution of the analyzed SNPs did not influence the overall survival rate of LSCC patients.

## Figures and Tables

**Table 1:** Demographic characteristics of the study population.

Cases (*n* = 200) age median (min–max) in years	64.5 (49–85) *
Controls (*n* = 200) age median (min–max) in years	65.5 (50–75) *
T1-2, *n* (%)	102 (51.0)
T3-4, *n* (%)	98 (49.0)
N0, *n* (%)	134 (67.0)
N1-3, *n* (%)	66 (33.0)
M0, *n* (%)	197 (98.5)
M1, *n* (%)	3 (1.5)
G1-2, *n* (%)	166 (83.0)
G3, *n* (%)	34 (17.0)
ST I-II, *n* (%)	97 (48.5)
ST III-IV, *n* (%)	103 (51.5)
* *p* = 0.073	

Abbreviations: T1-2—small tumor, T3-4—large tumor, N0—lymph nodes without metastasis, N1-3—lymph nodes with metastasis, M0—no distant metastasis to other organs, M1—distant metastasis present, ST I-II—early clinical stage, ST III-IV—advanced clinical stage, G1-2—well/moderately differentiated cancers, G3—poorly differentiated cancers.

**Table 2:** Genotype and allelic frequency distribution among case and control subjects, and their association with LSCC risk. (Bold text highlighting statistically significant results).

Gene	Polymorphism	Genotype/Allele	Frequency		*p* Value	OR (95% CI)	*p* Value	aOR (95% CI)	*p* Value
			Controls (*n* = 200)	Cases (*n* = 200)					
*TP53*	rs9895829	AA	174 (87.0)	178 (89.0)	0.060	Reference		Reference	
		**AG**	**13 (6.5)**	**4 (2.0)**		**0.301 (0.096–0.940)**	**0.039**	**0.300 (0.096–0.939)**	**0.039**
		GG	13 (6.5)	18 (9.0)		1.354 (0.644–2.846)	0.425	1.377 (0.653–2.903)	0.400
		AG + GG	26 (13.0)	22 (11.0)	0.645	0.827 (0.452–1.515)	0.539	0.835 (0.455–1.531)	0.559
		A	361 (90.3)	360 (90.0)	1.000	Reference		Reference	
		G	39 (9.8)	40 (10.0)		1.028 (0.646–1.637)	0.906	1.041 (0.654–1.659)	0.865
	rs17884306	CC	176 (88.0)	178 (89.0)	0.752	Reference		Reference	
		CT	24 (12.0)	21 (10.5)		0.865 (0.465–1.611)	0.648	0.866 (0.465–1.615)	0.652
		TT	0 (0.0)	1 (0.5)		not applicable		not applicable	
		CT + TT	24 (12.0)	22 (11.0)	0.876	0.906 (0.490–1.676)	0.754	0.912 (0.493–1.689)	0.770
		C	376 (94.0)	377 (94.3)	1.000	Reference		Reference	
		T	24 (6.0)	23 (5.8)		0.956 (0.530–1.723)	0.880	0.967 (0.536–1.745)	0.910
*BAX*	rs704243	AA	126 (63.0)	142 (71.0)	0.227	Reference		Reference	
		AG	68 (34.0)	54 (27.0)		0.705 (0.458–1.084)	0.111	0.703 (0.457–1.081)	0.109
		GG	6 (3.0)	4 (2.0)		0.592 (0.163–2.144)	0.424	0.605 (0.166–2.198)	0.445
		AG + GG	74 (37.0)	58 (29.0)	0.111	0.695 (0.457–1.058)	0.089	0.695 (0.457–1.057)	0.089
		A	320 (80.0)	338 (84.5)	0.115	Reference		Reference	
		G	80 (20.0)	62 (15.5)		0.734 (0.509–1.057)	0.097	0.735 (0.510–1.059)	0.098
*NOXA*	rs78800940	GG	98 (49.0)	100 (50.0)	0.808	Reference		Reference	
		GA	79 (39.5)	81 (40.5)		1.005 (0.662–1.524)	0.982	1.014 (0.668–1.541)	0.946
		AA	23 (11.5)	19 (9.5)		0.810 (0.415–1.580)	0.536	0.793 (0.405–1.550)	0.497
		AA + AG	102 (51.0)	100 (50.0)	0.920	0.961 (0.649–1.422)	0.841	0.964 (0.651–1.427)	0.853
		G	275 (68.8)	281 (70.3)	0.701	Reference		Reference	
		A	125 (31.3)	119 (29.8)		0.932 (0.689–1.259)	0.645	0.928 (0.687–1.255)	0.629
	rs1041978	GG	161 (80.5)	155 (77.5)	0.780	Reference		Reference	
		GT	35 (17.5)	41 (20.5)		1.217 (0.736–2.010)	0.444	1.260 (0.759–2.090)	0.372
		TT	4 (2.0)	4 (2.0)		1.039 (0.255–4.226)	0.958	1.064 (0.260–4.346)	0.931
		GT + TT	39 (19.5)	45 (22.5)	0.540	1.199 (0.740–1.941)	0.462	1.239 (0.762–2.016)	0.387
		G	357 (89.3)	351 (87.8)	0.580	Reference		Reference	
		T	43 (10.8)	49 (12.3)		1.159 (0.750–1.791)	0.506	1.192 (0.769–1.845)	0.432
*BCL2*	rs1564483	CC	107 (53.5)	111 (55.5)	0.394	Reference		Reference	
		CT	80 (40.0)	70 (35.0)		0.843 (0556–1.279)	0.423	0.845 (0.557–1.282)	0.427
		TT	13 (6.5)	19 (9.5)		1.409 (0.663–2.994)	0.373	1.376 (0.646–2.932)	0.408
		CT + TT	93 (46.5)	89 (44.5)	0.763	0.923 (0.622–1.368)	0.688	0.919 (0.620–1.364)	0.676
		C	294 (73.5)	292 (73.0)	0.936	Reference		Reference	
		T	106 (26.5)	108 (27.0)		1.026 (0.750–1.403)	0.873	1.019 (0.744–1.394)	0.907
	rs4987855	CC	178 (89.0)	179 (89.5)	0.871	Reference		Reference	
		CT	22 (11.0)	20 (10.0)		0.904 (0.477–1.715)	0.757	0.900 (0.474–1.709)	0.749
		TT	0 (0.0)	1 (0.5)		not applicable		not applicable	
		CT + TT	22 (11.0)	21 (10.5)	1.000	0.949 (0.504–1.787)	0.872	0.943 (0.500–1.777)	0.856
		C	378 (94.5)	378 (94.5)	1.000	Reference		Reference	
		T	22 (5.5)	22 (5.5)		1.000 (0.544–1.837)	1.000	0.991 (0.539–1.822)	0.977

*n*—number of patients, OR—odds ratios, CI—confidential interval, aOR—adjusted odds ratio; The aORs were estimated with multivariate logistic regression analysis after adjustment for age.

**Table 3:** Genotype and allelic association with patient age at diagnosis. (Bold text highlighting statistically significant results).

SNP	Genotype/Allele		Frequency		*p* Value	OR (95% CI)	*p* Value
*NOXA* rs1041978	GG	*n* (%)	96 (83.5)	59 (69.4)	Reference		
	**GT**	***n* (%)**	**17 (14.8)**	**24 (28.2)**	**2.297**	**1.140–4.630**	**0.020**
	TT	*n* (%)	2 (1.7)	2 (2.4)	1.627	0.223–11.863	0.631
	*p* value		0.062				
	GG	*n* (%)	96 (83.5)	59 (69.4)	Reference		
	**TT + GT**	***n* (%)**	**19 (16.5)**	**26 (30.6)**	**2.227**	**1.134–4.371**	**0.020**
	*p* value		0.025				
	G allele	*n* (%)	209 (90.9)	142 (83.5)	Reference		
	**T allele**	***n* (%)**	**21 (9.1)**	**28 (16.5)**	**1.962**	**1.072–3.592**	**0.029**
	*p* value		**0.031**				

## Data Availability

The datasets used and analyzed during the current study are available from the corresponding author on request.

## Supplementary Materials

The following supporting information can be downloaded at: https://www.mdpi.com/article/10.3390/ijms252111849/s1.
