# Supplementary material for: The Association of TP53, BCL2, BAX and NOXA SNPs and Laryngeal Squamous Cell Carcinoma Development
Source: Int J Mol Sci. 2024 Nov 4;25(21):11849. doi: 10.3390/ijms252111849 (PMC11546907; doi:10.3390/ijms252111849)
Supplement: Supplementary file 1 [file ijms-25-11849-s001.zip › ijms-3236754-supplementary.pdf]

Supplementary figure S1. Overall survival rate according to the distribution of *TP53* rs9895829 genotypes.

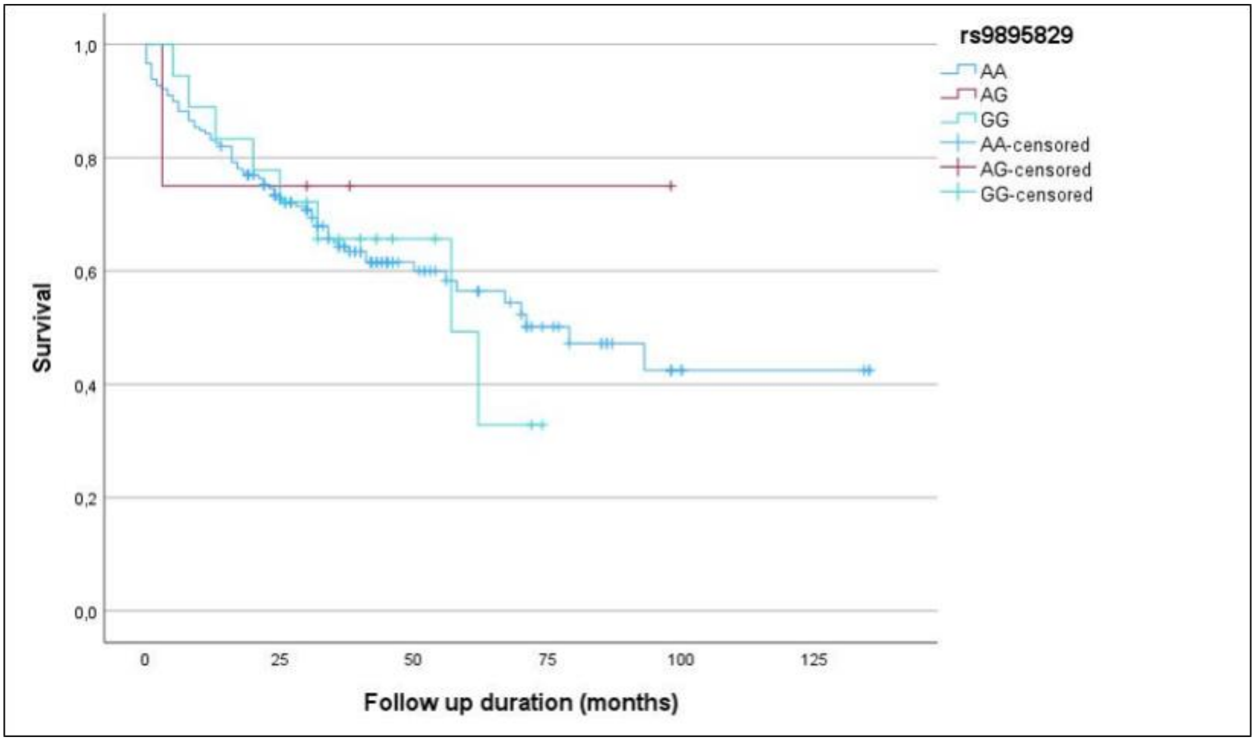

Supplementary table S1. Log-rank, Breslow and Tarone-Ware tests for overall survival rate comparison according to the distribution of *TP53* rs9895829 genotypes.

	Chi-Square	df	p-value
Log Rank (Mantel-Cox)	0.366	2	0.833
Breslow (Generalized Wilcoxon)	0.069	2	0.966
Tarone-Ware	0.112	2	0.946

Supplementary figure S2. Overall survival rate according to the distribution of *TP53* rs17884306 genotypes.

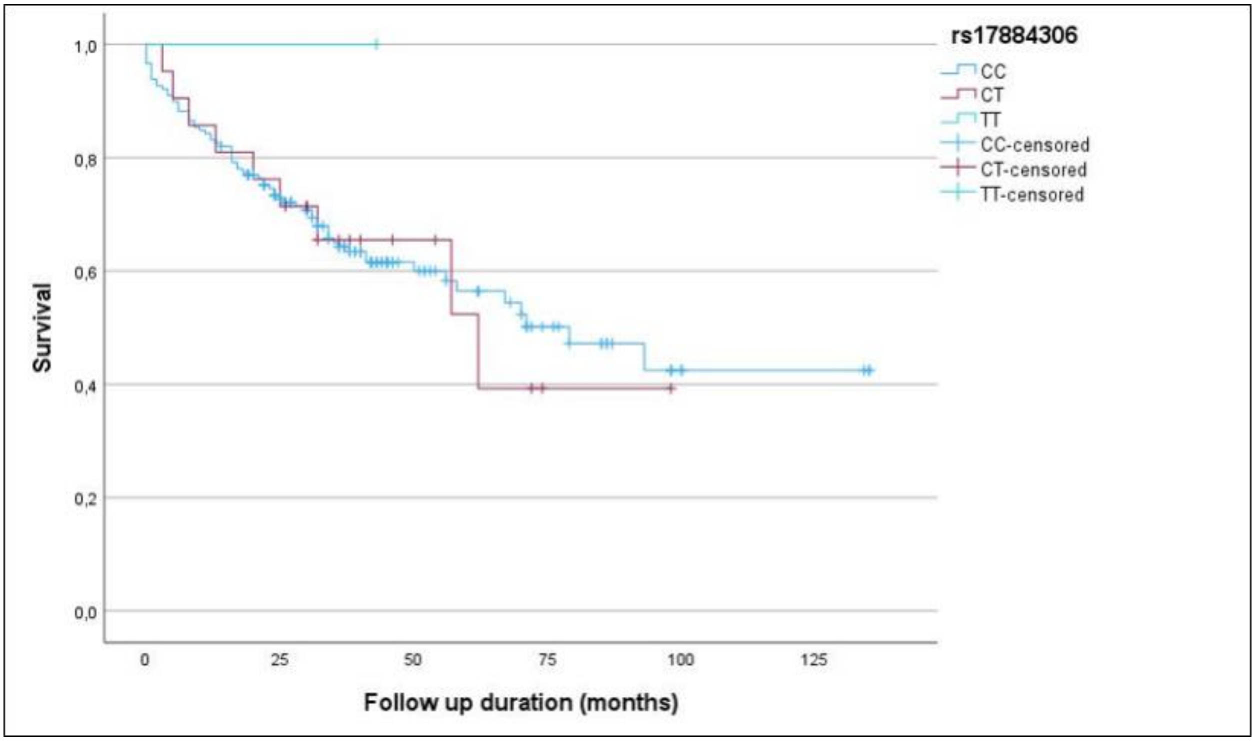

Supplementary table S2. Log-rank, Breslow and Tarone-Ware tests for overall survival rate comparison according to the distribution of *TP53* rs17884306 genotypes.

	Chi-Square	df	p-value
Log Rank (Mantel-Cox)	0.500	2	0.779
Breslow (Generalized Wilcoxon)	0.463	2	0.793
Tarone-Ware	0.472	2	0.790

Supplementary figure S3. Overall survival rate according to the distribution of *BAX* rs704243 genotypes.

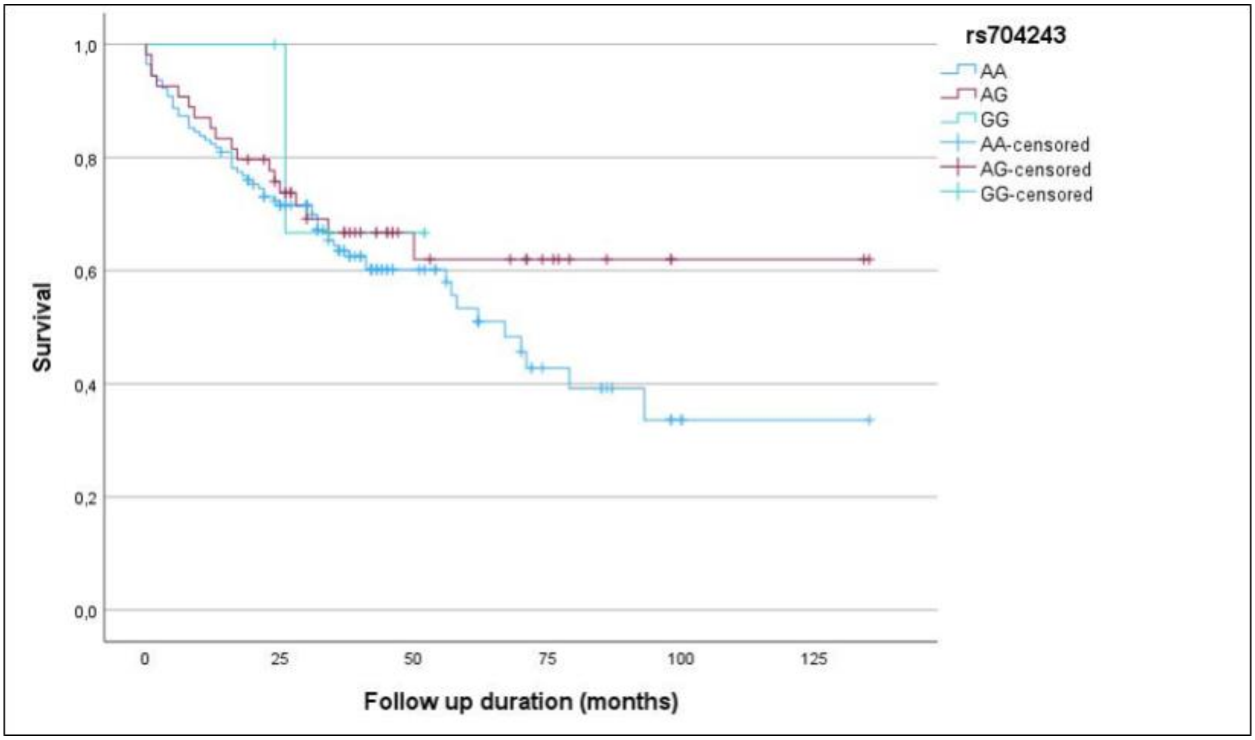

Supplementary table S3. Log-rank, Breslow and Tarone-Ware tests for overall survival rate comparison according to the distribution of *BAX* rs704243 genotypes.

	Chi-Square	df	p-value
Log Rank (Mantel-Cox)	1.659	2	0.436
Breslow (Generalized Wilcoxon)	0.745	2	0.689
Tarone-Ware	0.949	2	0.622

Supplementary figure S4. Overall survival rate according to the distribution of *NOXA* rs78800940 genotypes.

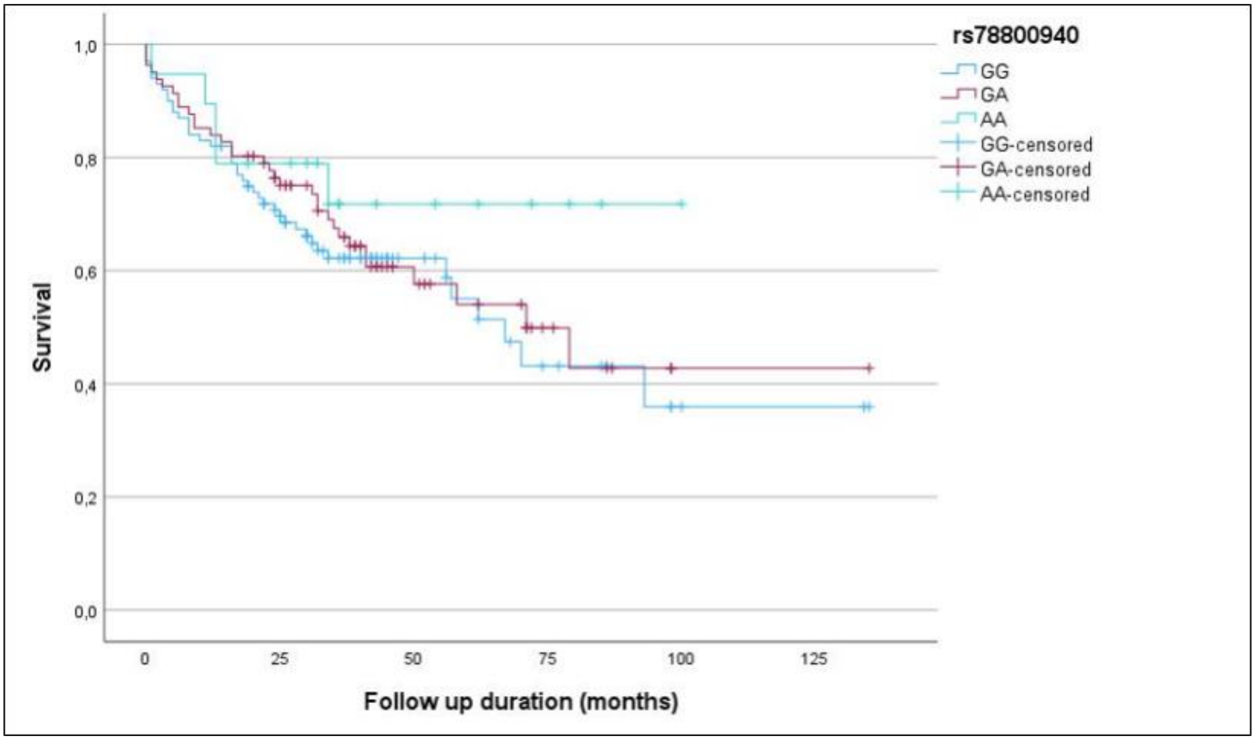

Supplementary table S4. Log-rank, Breslow and Tarone-Ware tests for overall survival rate comparison according to the distribution of *NOXA* rs78800940 genotypes.

	Chi-Square	df	p-value
Log Rank (Mantel-Cox)	1.701	2	0.427
Breslow (Generalized Wilcoxon)	1.045	2	0.593
Tarone-Ware	1.233	2	0.540

Supplementary figure S5. Overall survival rate according to the distribution of *NOXA* rs1041978 genotypes.

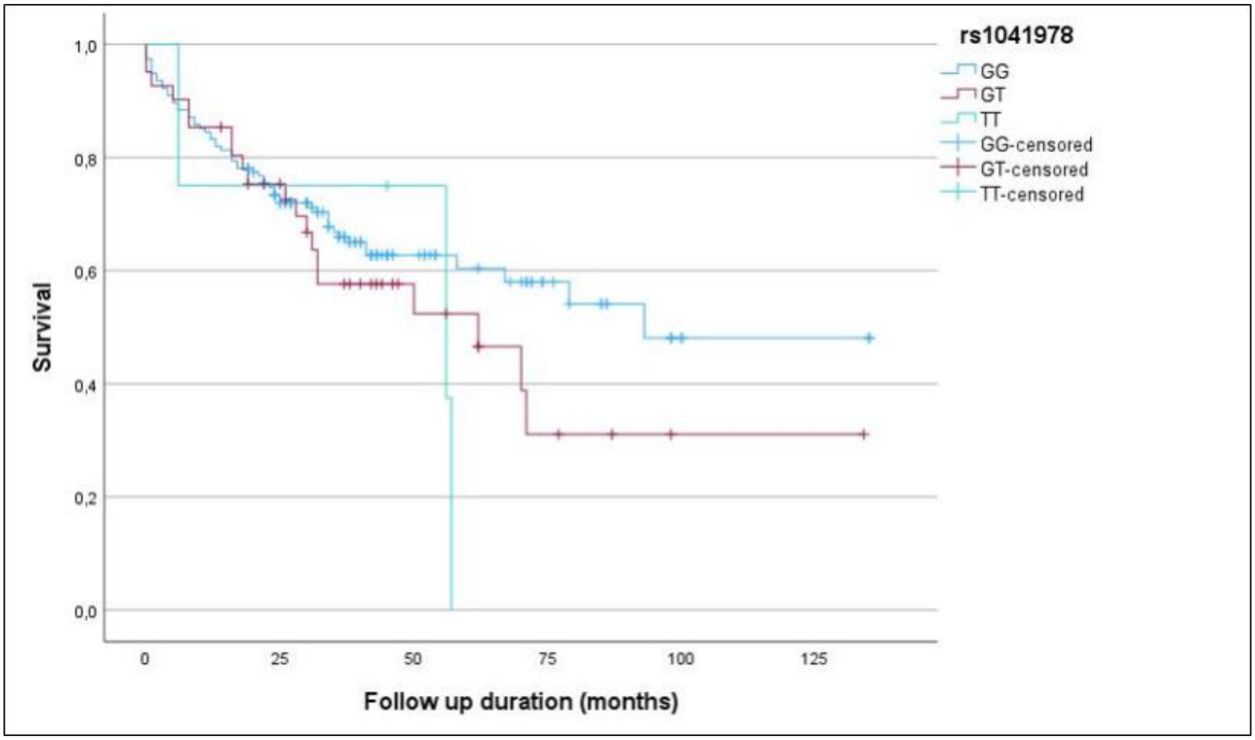

Supplementary table S5. Log-rank, Breslow and Tarone-Ware tests for overall survival rate comparison according to the distribution of *NOXA* rs1041978 genotypes.

	Chi-Square	df	p-value
Log Rank (Mantel-Cox)	2.339	2	0.311
Breslow (Generalized Wilcoxon)	0.337	2	0.845
Tarone-Ware	0.786	2	0.675

Supplementary figure S6. Overall survival rate according to the distribution of *BCL2* rs1564483 genotypes.

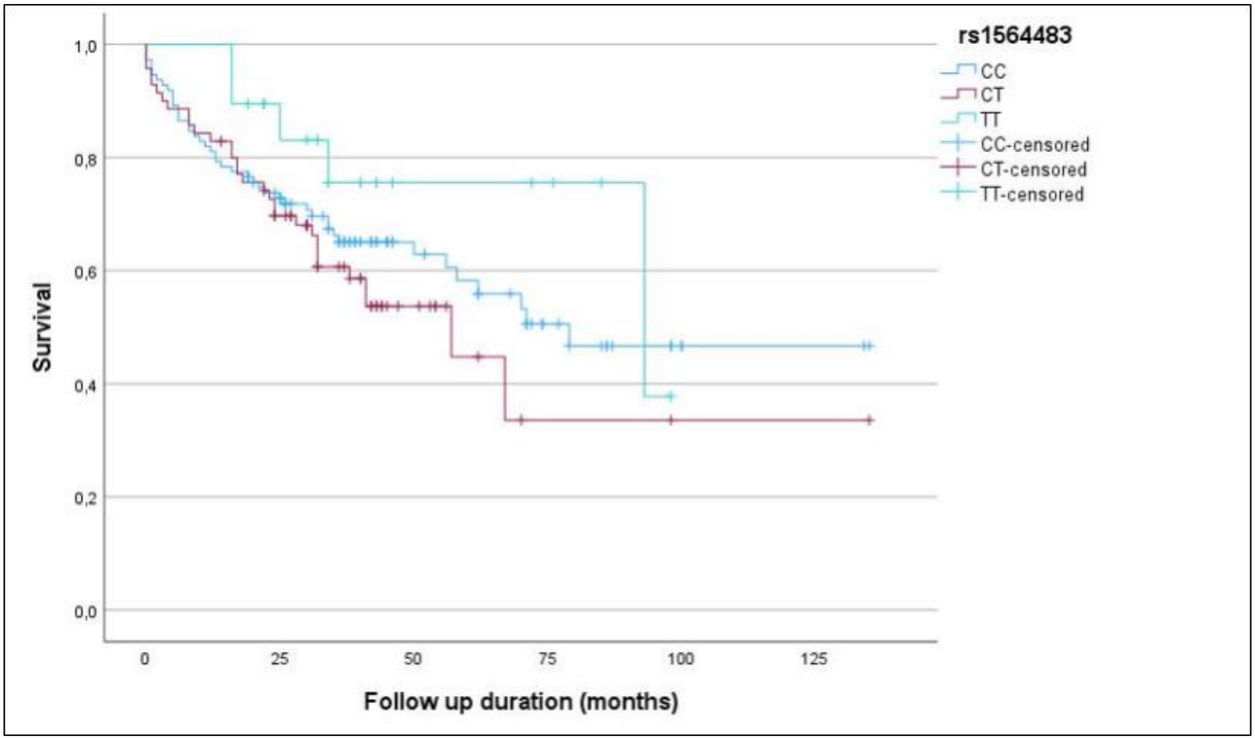

Supplementary table S6. Log-rank, Breslow and Tarone-Ware tests for overall survival rate comparison according to the distribution of *BCL2* rs1564483 genotypes.

	Chi-Square	df	p-value
Log Rank (Mantel-Cox)	3.128	2	0.209
Breslow (Generalized Wilcoxon)	2.783	2	0.249
Tarone-Ware	3.021	2	0.221

Supplementary figure S7. Overall survival rate according to the distribution of *BCL2* rs4987855 genotypes.

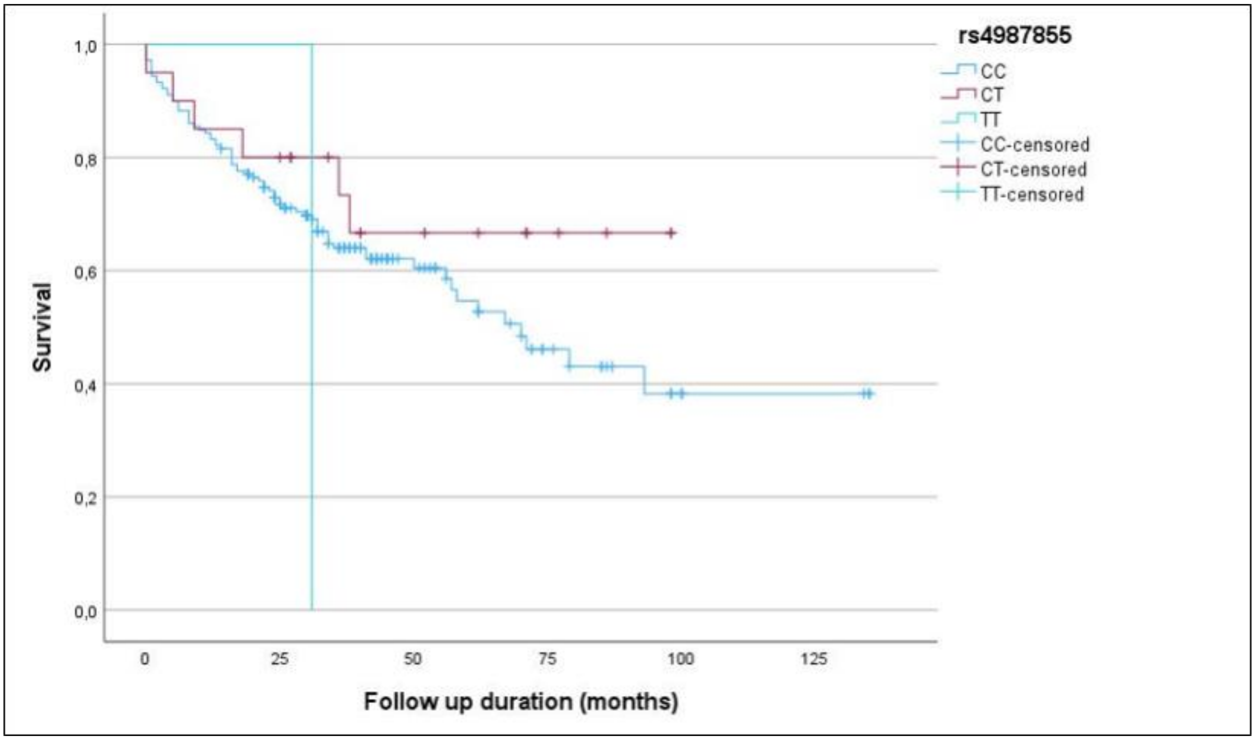

Supplementary table S7. Log-rank, Breslow and Tarone-Ware tests for overall survival rate comparison according to the distribution of *BCL2* rs4987855 genotypes.

	Chi-Square	df	p-value
Log Rank (Mantel-Cox)	2.511	2	0.285
Breslow (Generalized Wilcoxon)	0.899	2	0.638
Tarone-Ware	1.459	2	0.482
